# Study of CuO Nanowire Growth on Different Copper Surfaces

**DOI:** 10.1038/s41598-018-37172-8

**Published:** 2019-01-28

**Authors:** Gerhard Fritz-Popovski, Florentyna Sosada-Ludwikowska, Anton Köck, Jozef Keckes, Günther A. Maier

**Affiliations:** 1 Materials Center Leoben GmbH, Roseggerstr. 12, 8700 Leoben, Austria; 2 Institute of Physics, Montanuniversität Leoben, 8700 Leoben, Austria; 3 Department of Materials Physics, Montanuniversität Leoben, 8700 Leoben, Austria

## Abstract

Cupric oxide (CuO) nanowires were produced by thermal oxidation of copper surfaces at temperatures up to 450 °C. Three different surfaces, namely a copper foil as well as evaporation deposited copper and an application relevant sputtered copper film on Si(100) substrates were characterized *ex-situ* before and after the experiment. The development of oxide layers and nanowires were monitored *in-situ* using grazing incidence small angle X-ray scattering. The number density of nanowires is highest for the sputtered surface and lowest for the surface prepared by evaporation deposition. This can be linked to different oxide grain sizes and copper grain boundary diffusions on the different surfaces. Small grains of the copper substrate and high surface roughness thereby lead to promoted growth of the nanowires.

## Introduction

In the recent years, metal oxide nanostructures have neem investigated for a variety of applications such as sensors, energy efficient coatings, and semiconductor devices^1–5^. Copper oxide nanowires have attracted considerable interest during the last couple of years. This is partly caused by the semiconductive properties of CuO having a bandgap of 1.85 eV^6^. The large surface of the nanowires is of advantage for many applications such as solar cells^7^, catalysis^8^, or sensor applications^9–12^.

Copper oxide nanowires can be grown by a wide variety of techniques^13^. Growing them by just heating a copper surface in oxygen containing atmosphere might be the simplest procedure^14–18^. The nanowires form on the surface at temperatures ranging from 300 °C to 700 °C. Typically, higher temperatures lead to an increase in diameter, while longer growing times lead to an increase in aspect ratio^19^.

The formation of the nanowires is connected to the two stable oxides of copper^20^, namely cuprous oxide (Cu_2_O) and cupric oxide (CuO). The copper surface is oxidied at 70–110 °C forming a layer of Cu_2_O or its defect structure Cu_3_O_2_^21^. Further heating to 200–270 °C leads to the formation of an additional layer of CuO on top of the Cu_2_O layer. The Cu_2_O layer is typically thicker than the CuO layer^22,23^ and the crystallites in the Cu_2_O layer are also larger than those in the CuO one^24^.

Crystal size of Cu and its oxides is, however, of great importance for the growth of the nanowires, which can form on top of CuO crystallites. In order to grow, copper atoms have to migrate through the two oxide films and up the nanowires. Grain boundary diffusion is considered to be the dominating contribution to this mass flow, followed by surface diffusion once the surface between the CuO grains have been reached^25,26^. Consequently, a larger number of grain boundaries leads to increased diffusion and therefore to better growth of the nanowires.

The metallic copper underneath can affect the growth of the nanowires indirectly. The increase in volume during oxidation and the corresponding lattice mismatch of the two phases leads to stresses at the copper-Cu_2_O interface. The different thermal expansion of a substrate and a copper film can also induce stresses which influence nanowire growth^27^. It has been shown that bending of copper foils leads to larger oxide grains at the compressed side and smaller on the tensile side. This reduces nanowire growth at the side of compressive stress and promotes it at the side of tensile stress^24^. Similarly, increasing the surface roughness of the copper substrate leads to the nucleation of more grains, which becomes manifest in an increased number of nanowires on such a surface^28^.

The aim of this work is to further elucidate the influence of the Cu substrate microstructure on the growth of cupric oxide nanowires by thermal oxidation. Therefore, three different Cu surfaces are investigated in terms of nanowire growth, namely a copper foil and two Cu films, evaporated and sputtered on silicon wafers. The motivation was to elucidate the growth process and microstructure of CuO nanowires on surfaces, which are commonly used in sensor applications. The silicon wafers had been coated with a titanium film as a undercoating^29^. All surfaces are characterized before thermal oxidation using atomic force microscopy (AFM) and electron backscattering diffraction (EBSD). The actual heating and oxidation process was monitored *in-situ* using grazing incidence small angle x-ray scattering (GISAXS). The final state was investigated using scanning electron microscopy (SEM). The phases present within the samples were also checked *ex-situ* by means of x-ray diffraction (XRD).

## Experimental

Three different copper surfaces were investigated. Copper foil (≥99.8%, 0.1 mm thick, Sigma-Aldrich) was washed in 1 M HCl/H_2_O after delivery. The “evaporated Cu” was prepared by evaporation of 5 nm Ti adhesion layer followed by thermal thin film vacuum metal deposition of 550 nm Cu (UNIVEX 450, Leybold AG (Päffikon, Switzerland)). The third sample, sputtered Cu, was prepared by sputtering of 5 nm thick Ti adhesion layer and then Cu layer of 750 nm using a Sigma® fxP PVD System (Standard PVD – conventional sputter module). All samples were cleaned before oxidation with isopropanol in ultrasonic bath for 3 min.

Surface topography was determined using a DME-BRR 2770 scanning probe microscope (SEMILAB-DME, Dk). The measurements were performed in non-contact mode using tips with a radius below 10 nm (Tap300Al-G, Budgetsensors).

Scanning electron microscopy (SEM) and electron backscatter diffraction (EBSD) analyses were performed using an Auriga cross beam workstation (Zeiss, D). It is equipped with a field emission electron source and an EBSD Detector (Oxford Instruments, GB). The dimension and the lateral resolution of the EBSD scans were choses to fit the grain size of the samples. Cross sections of the films were cut directly in the instruments with a focused ion beam source using gallium ions. Prior the focused ion beam (FIB) milling a Pt protection layer was deposited inside the microscope by ion beam assisted chemical vapour deposition from metal organic precursor.

X-Ray Diffraction (XRD) experiments were performed using a 5 circle diffractometer SmartLab (Rigaku, J) with Cu-Kα radiation, a primary parabolic multilayer mirror and a secondary graphite monochromator. A symmetric diffraction scan was measured using a 2θ measurement step of 0.02 deg and a speed of 2 deg/minute.

Grazing incidence small angle scattering experiments were performed using a NANOSTAR instrument (Bruker, Germany) equipped with an IµS microsource and a VÅNTEC-2000 detector operating at a wavelength of 1.54 Å (Cu Kα). The heating stage was based on a TC-DOME (Bruker, Germany) and placed within the evacuated sample chamber. A controlled atmosphere was provided by means of a double shelled cooled aluminium dome having an entry and an exit window made of Kapton™^30^. The dome was connected to the exterior air and a continuous flow of fresh air was guaranteed. The temperature was measured using a S-type thermocouple in contact with the specimen and controlled by means of a TCU1 control unit (Bruker, Germany). The incidence angle of the primary beam was set to 0.25°.

All types of samples were simultaneously oxidized and measured using GISAXS using two different heat treatments. First they were heated at a rate of 10 °C per minute from room temperature to 450 °C followed by two hours at a constant temperature of 450 °C. GISAXS patterns with an integration time of 10 minutes were collected at room temperature and repeatedly during the final constant temperature step. During ramping up patterns were recorded every minute. The second series consisted of temperature steps at 25, 100, 125, 150, 160, 170, 180, 190, 200, 210, 220, 230, 240, 250, 275, 300, 325, 350, 375, 400, 425, 450 °C. The sample was kept at the specified temperatures for about 10 minutes before the GISAXS pattern was collected for another 10 minutes. The sample detector distance was calibrated by a measurement of silver behenate^31^.

Horizontal cuts *I*(*q*) parallel to the surface were taken from the two dimensional GISAXS scattering, where $q=\sqrt{{q}_{x}^{2}+{q}_{y}^{2}}$ and

$${\bf{q}}=(\begin{array}{c}{q}_{x}\\ {q}_{y}\\ {q}_{z}\end{array})=\frac{2\pi }{\lambda }(\begin{array}{c}\cos \,{\alpha }_{f}\,\cos \,{\vartheta }_{f}-\,\cos \,{\alpha }_{i}\\ \cos \,{\alpha }_{f}\,\sin \,{\vartheta }_{f}\\ \sin \,{\alpha }_{f}+\,\sin \,{\alpha }_{i}\end{array})$$

is the scattering vector. Here *λ* is the wavelength and *α*_*i*_ is the incidence angle. The angles ${\vartheta }_{f}$ and *α*_*f*_ are the angles defining the scattered beam in horizontal and vertical direction. A parameter related to the correlation length^26^ was computed from these cuts as

$${L}_{c}=\pi \frac{{\int }_{{q}_{1}}^{{q}_{2}}qI(q)dq}{{\int }_{{q}_{1}}^{{q}_{2}}{q}^{2}I(q)dq}$$

where *q*_1_ = 0.1 nm^−1^ and *q*_2_ is the experimentally accessible upper limit of the *q*-scale. In addition, the scattering curves were approximated by *I*(*q*) = *aq*^*α*^ + *b*. This approximation was done within the *q*-range larger than 0.112 nm^−1^ for the copper foil and larger than 0.1 for the other samples and can be seen as a generalization of the Porod law^32^.

## Results

**Figure 1:**
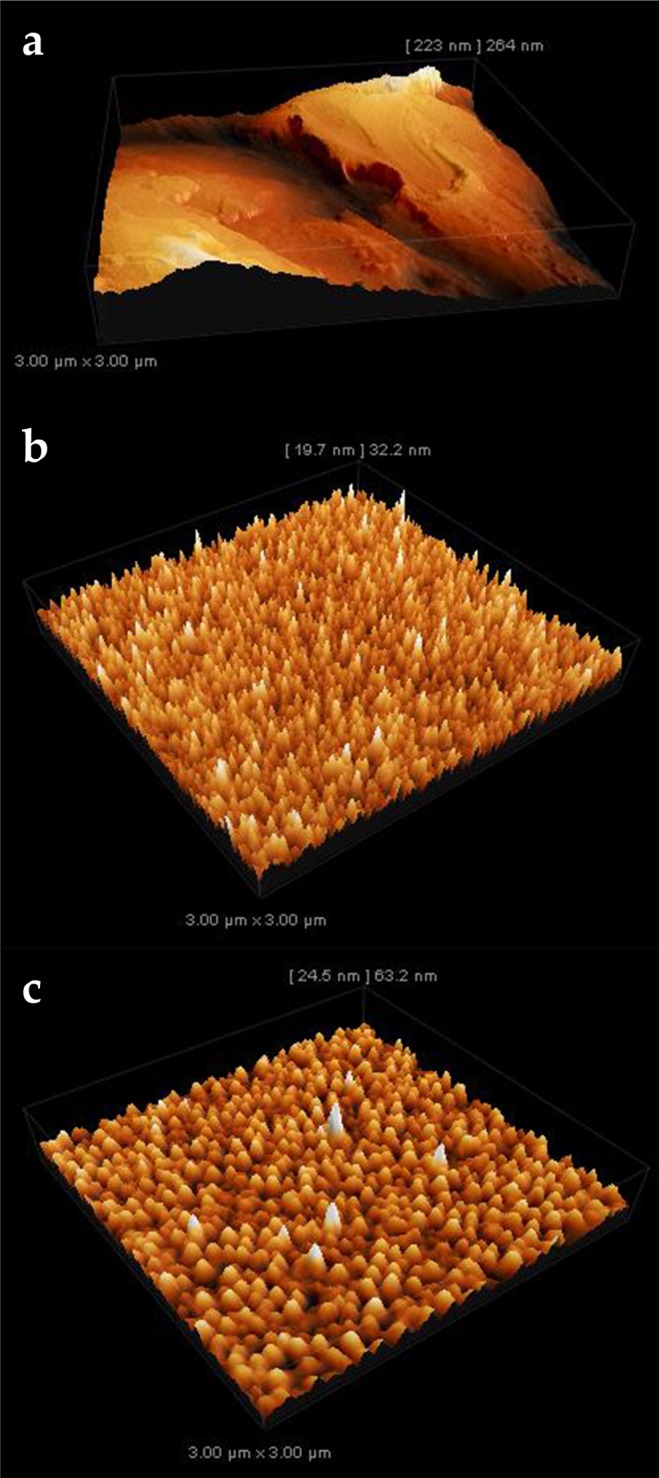
Atomic force microscopy measurements of the initial (**a**) copper foil, (**b**) copper film deposited from evaporated copper, and (**c**) film prepared from sputtered copper. The areas shown are 3 × 3 µm^2^ large and the scale shows the maximum height difference in the scanned area.

Atomic force microscopy measurements of the three surfaces are shown in Fig. 1 (Supporting information Fig. S1). In the case of the copper foil a large roughness average (Ra) value of 48 nm and a maximum height difference from 260 nm was determined. Evaporated copper film has a very small roughness average of 3.1 nm showing a great number of pyramidal surface features. No preferential orientation is visible, which is also the case for sputtered copper. In this sample, a slightly higher Ra value of 4.5 nm was determined. The lateral size of the more dome shaped features is also larger as for the film prepared by evaporating copper.

**Figure 2:**
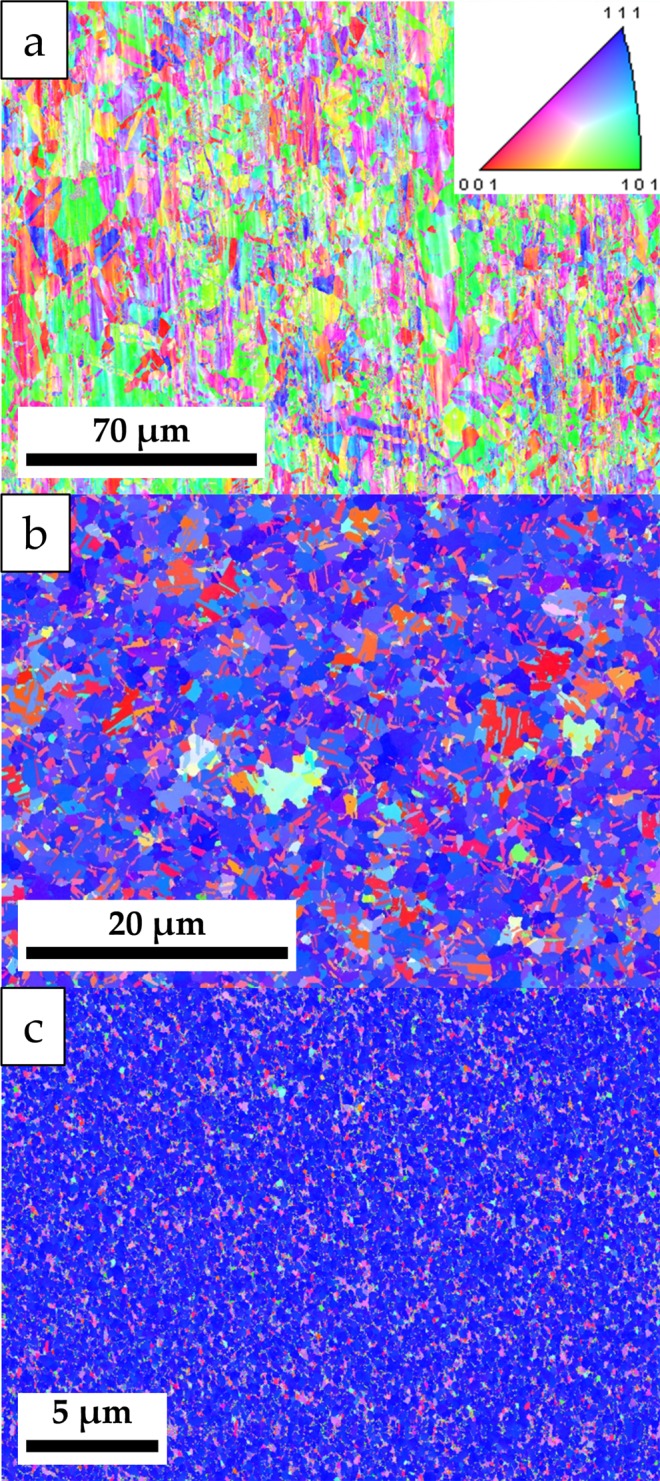
Inverse pole figure maps based on electron backscattering diffraction of the (**a**) copper foil, (**b**) copper film deposited from evaporated copper, and (**c**) film prepared from sputtered copper. The insert in (**a**) gives the colour code of the images.

Inverse pole figure maps obtained via EBSD (Fig. 2) show laterally large grains on the surface of the copper foil, although some consist of much smaller grains (Supporting information, Fig. S2). The average grain size is found to be 4.2 ± 3.4 µm and few of the grains exhibit a {111} surface. This is contrary to the observations made for the other two surfaces, where the {111} surfaces of the grains dominate the EBSD inverse pole figure maps. The grains of the surface deposited with evaporation technique are considerably smaller than the ones found on the surface of the copper foil having a diameter of 1.12 ± 0.65 µm. Besides the {111} surfaces, there is a considerable amount of grains that show {001} surfaces. This is not observed for the grains on the surface prepared by sputtering of copper. In this case, basically all features on the surface show only the {111} orientation, while other orientations are only observed in the interstices between these features (Supporting information Fig. S3). The average diameter of these grains is 0.26 ± 0.13 µm.

**Figure 3:**
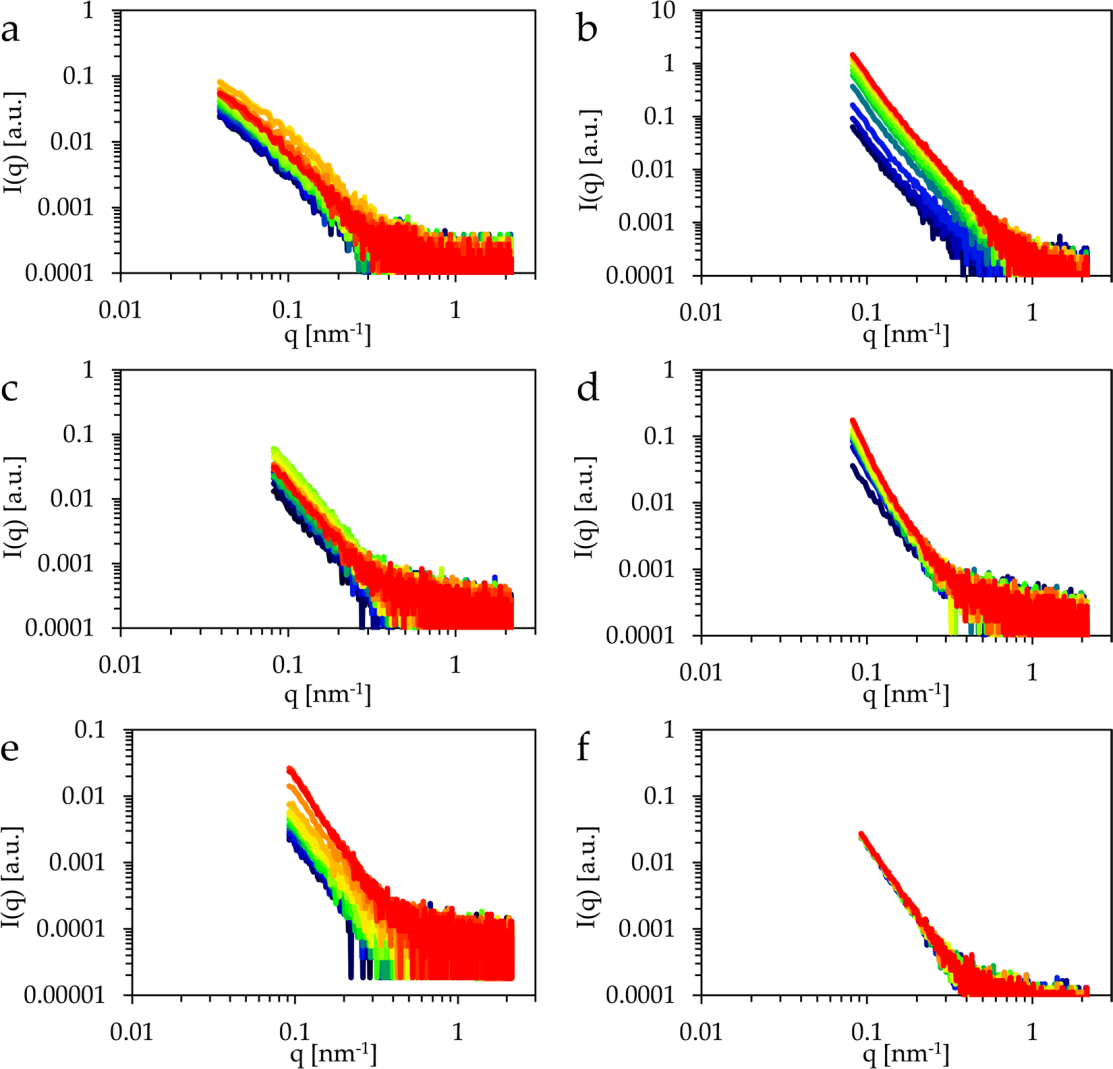
Scattering curves obtained from horizontal cuts through GISAXS scattering patterns of copper foil (**a**) during heating, (**b**) at constant 450 °C. Curves of the surface deposited from evaporated copper (**c**) during heating, (**d**) at constant 450 °C. Curves of the film prepared by sputtered copper (**e**) during heating and, (**f**) at constant 450 °C. The curves are coloured from blue to red with increasing temperature in (**a**), (**c**), and (**e**) and in the same order with increasing time in (**b**), (**d**), and (**f**).

The GISAXS data presented here for the temperature dependence are the ones obtained from the step wise heating series. The scattering curves obtained from GISAXS (Fig. 3) all show a monotonic decay with increasing scattering angle. The constant background is small in all cases.

The profiles that have been measured during the thermal treatment of the foil (Fig. 3a) increase in intensity up to about 350 °C and decrease when the sample is heated further. All curves show a weak shoulder in double logarithmic representation, but it is most pronounced at the temperatures that pertain to the highest intensities. The scattering curves of the same material at a constant temperature of 450 °C evince a pronounced increase of intensity with time (Fig. 3b).

GISAXS data of the copper surface deposited by evaporation show a basically constant slope without a shoulder during the heating process (Fig. 3c). Once again the curves reach an intensity maximum at intermediate temperatures, in this case at 160 °C. The series at constant 450 °C, however, is dominated by an increase in slope with time (Fig. 3d).

The scattering curves measured at the sputtered copper surface show an increase in intensity with temperature (Fig. 3e) that is combined with a steepening of the slope. The measurement at constant 450 °C shows no further significant change of the scattering profiles (Fig. 3f).

**Figure 4:**
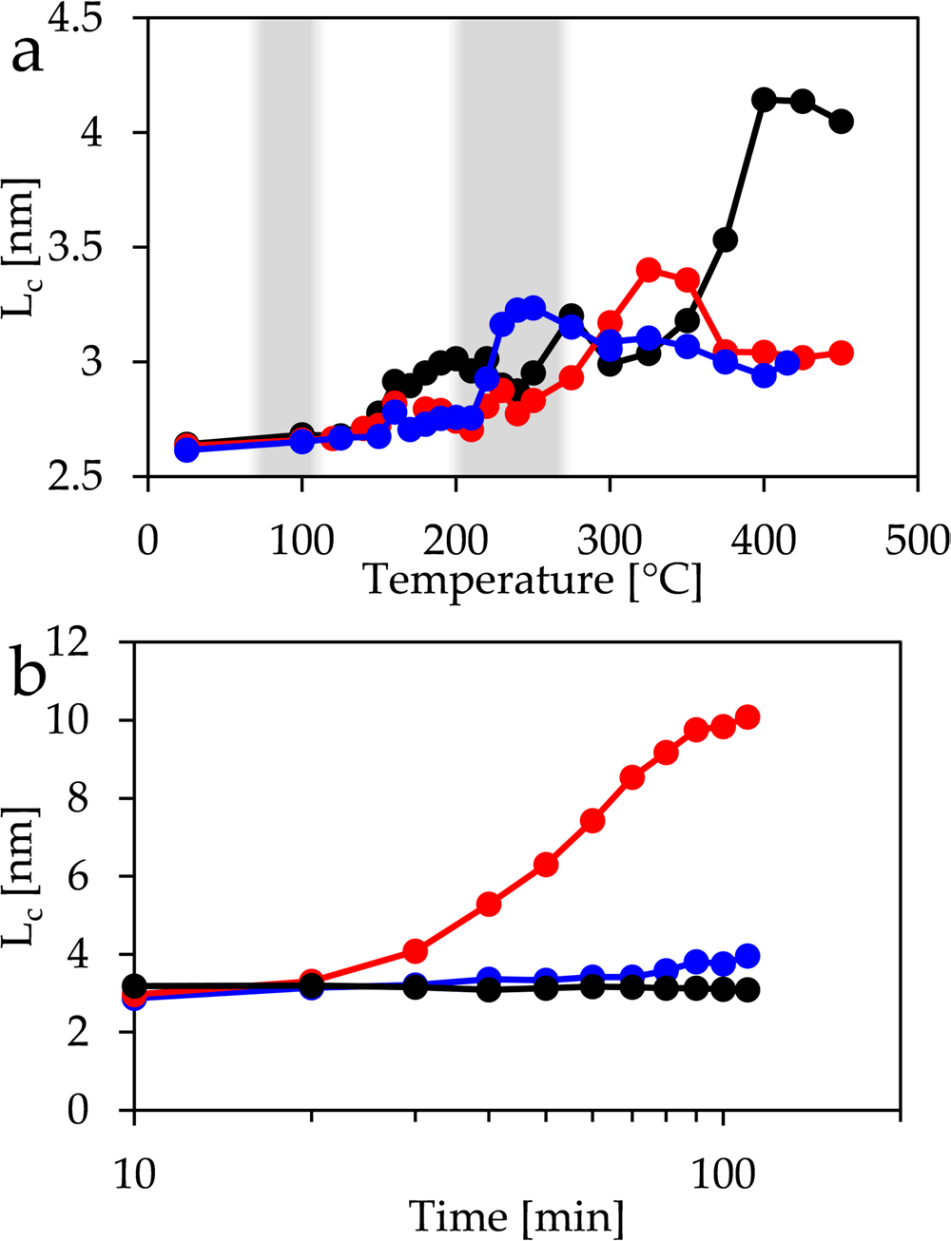
Development of parameter *L*_*c*_ with (**a**) temperature and (**b**) with time at 450 °C for copper foil (red), vapour deposited copper (blue) and sputtered copper (black). Oxidation temperatures to Cu_2_O and to CuO are shaded.

The parameter *L*_*c*_ obtained from the scattering profiles of the copper foil (Fig. 4) shows little change with temperature up to 275 °C. Thereafter, *i.e*. once the CuO film has fully developed, there is a first increase followed by a drop at 375 °C. Once the maximum temperature of 450 °C has been reached, *L*_*c*_ rises strongly with time. In the case of the film deposited from evaporated copper, there is a considerable increase of *L*_*c*_ at the temperatures that are typical for oxidation of Cu_2_O to CuO, followed by a slight decrease. Once a temperature of 450 °C has been reached, the parameter grows slightly with time. Finally, in the case of sputtered copper, *L*_*c*_ has also a maximum close to the temperatures where CuO forms. At temperatures above 300 °C *L*_*c*_ increases strongly and reaches at 400 °C its maximum, which basically does not change any more, even when kept at a constant temperature of 450 °C.

**Figure 5:**
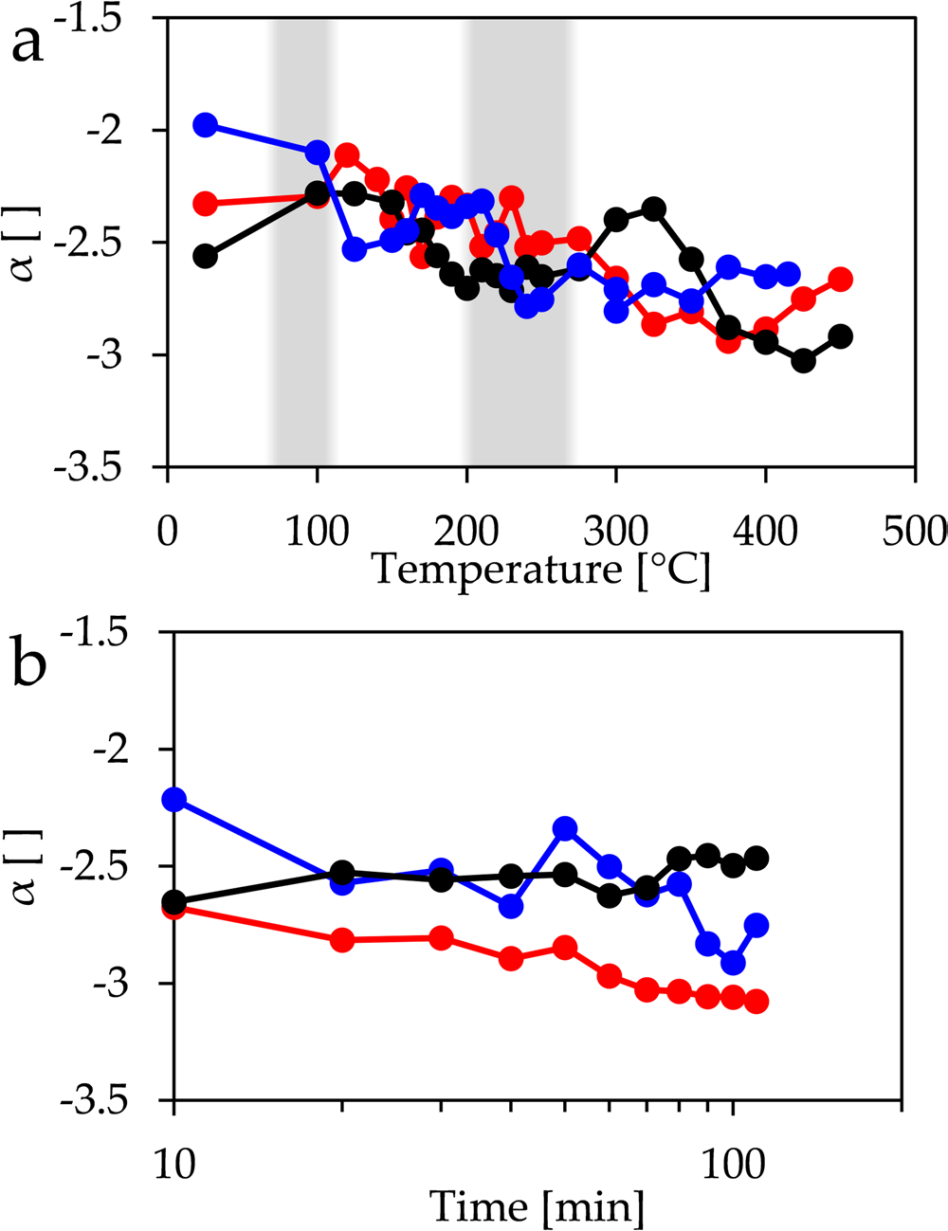
Development of the power law exponent α with (**a**) temperature and (**b**) with time at 450 °C for copper foil (red), vapour deposited copper (blue) and sputtered copper (black). Oxidation temperatures to Cu_2_O and to CuO are shaded.

The slope of the scattering profiles (Fig. 5) of the foil shows no clear trend up to the temperature, where CuO forms. At higher temperatures the slope is steeper, but remains basically constant, while keeping the sample at 450 °C leads to a gradual steepening of the profile. A similar behaviour can be observed for the film of evaporated copper, although there might be an additional step in slope at about 100 °C, where Cu_2_O forms. The sputtered copper surface shows, however, a steepening of the slope already at temperatures below 200 °C. A second step is observed at 300–400 °C, while no change is observed when the sample is kept at 450 °C.

**Figure 6:**
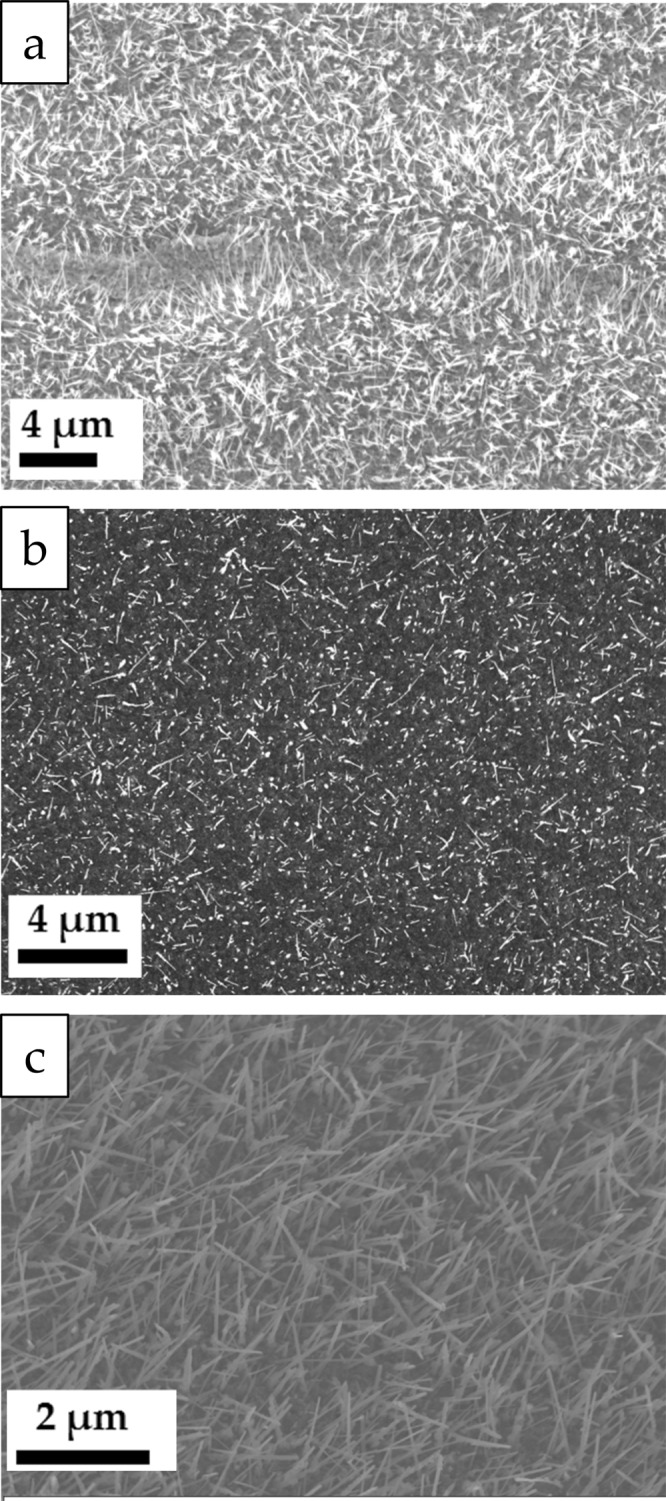
Scanning electron microscopy images of the (**a**) copper foil, (**b**) vapour deposited copper, and (**c**) sputtered copper after the heat treatment.

The SEM images of the three surfaces after thermal treatment (Fig. 6) show nanowire growth in all three cases, where the wires have a diameter of approximately 60 nm. The copper foil is covered in most places by nanowires with a number density of 4.9 ± 2.0 µm^−2^. One elongated valley-like feature does not show nanowires growing from within, but it seems to be filled with granular structures. The nanowires, on the other hand do not seem to originate uniformly from the surface, but preferably from some small spots. The length can only be estimated roughly, due to the projection visible, but is at least 2–7 µm as estimated from a Focused Ion Beam cut (Supporting information Fig. S12) on a random position. The evaporated copper that has been deposited as a film shows, after the thermal treatment, a low density of nanowires of 3.2 ± 1.7 µm^−2^. Several areas on the size of 1 × 1 µm² are even basically void of any grown structure. The nanowires that are present on this surface are also in most cases shorter than in the case of the copper foil having a projected length of 0.3–1 µm. Finally the surface of the sputtered copper film is covered by a thick layer of nanowires, which has a density of 16.3 ± 2.6 µm^−2^. These nanowires have a high aspect ratio with a visible length of at least 0.5–2 µm.

**Figure 7:**
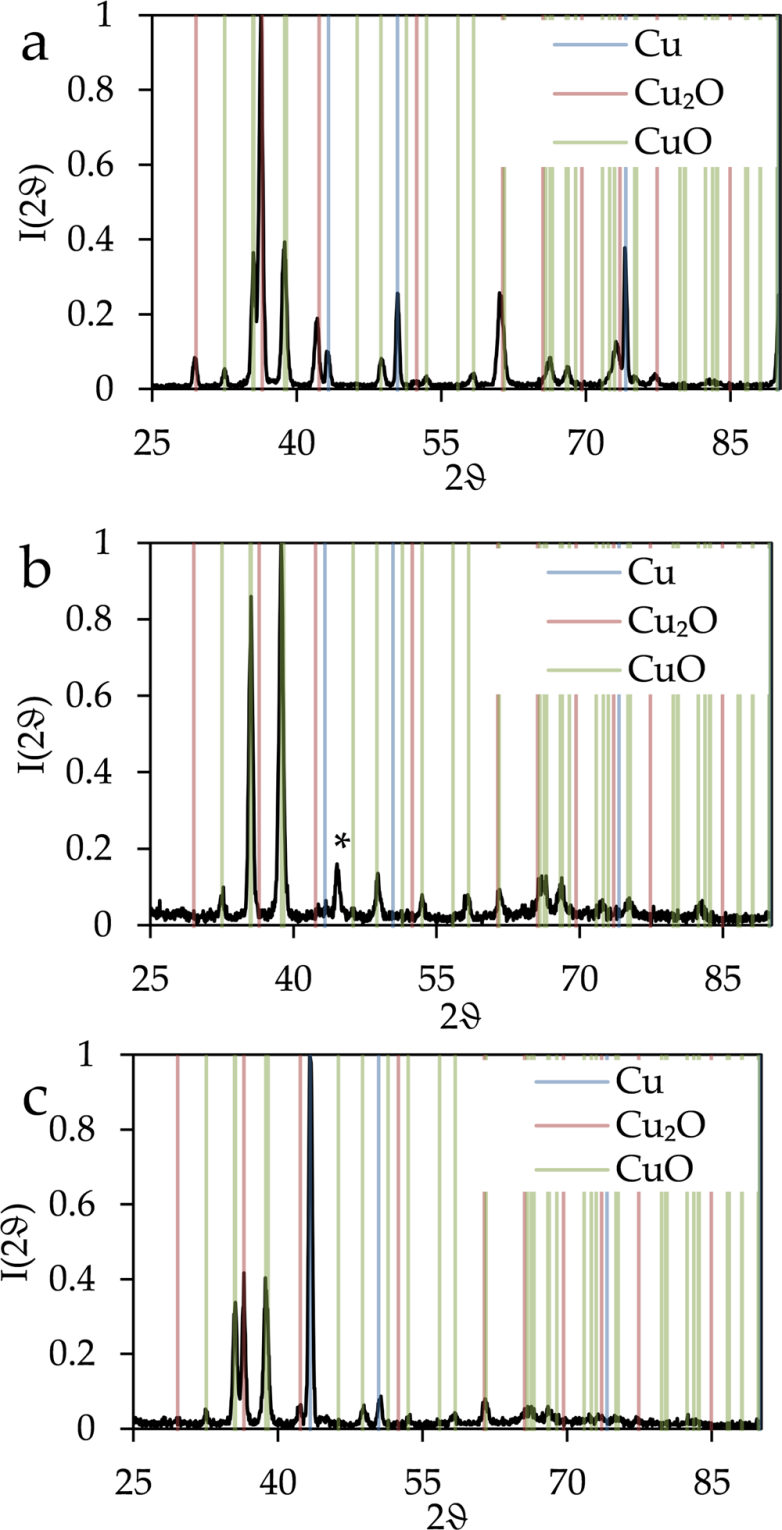
X-ray diffraction of (**a**) copper foil, (**b**) vapour deposited copper, and (**c**) sputtered copper after the heat treatment. The coloured lines indicate the theoretical peak positions of Cu, Cu_2_O and CuO. A peak due to Cu_3_Si is marked with a star.

X-ray diffraction results in spectra that show after thermal treatment peaks (Fig. 7) corresponding to metallic copper as well as to Cu_2_O and CuO. The spectra of copper foil shows peaks that can be attributed to all these three compounds: The copper (111) peak is seen at 43.2° and the copper (002) peak at 50.3° Similarly, the Cu_2_O (111) peak can be observed at 36.4° and the Cu_2_O (002) peakat 42.2°. CuO leads to overlapping (002) and $(\bar{1}11)$ peaks at 35.4° and 35.5° as well as to overlapping (111) and (200) peaks at 38.7° and 38.9°. The same peaks can also be found in the XRD pattern of the sample based on vapout deposited copper. However, the XRD spectrum of the sputtered copper surface is largely dominated by peaks that are due to CuO, and the mentioned peaks of metallic copper and Cu_2_O cannot be identified here, despite of the fact that they should the most prominent ones of these components. An additional peak found for this sample at 44.6° in the XRD spectrum can be identified as Cu_3_Si^33–35^. This indicates that the thermal treatment of Cu films on Si substrates resulted in the expected diffusion of Si into the films and the build-up of silicides at the interfaces. Therefore, a detailed quantitative analysis of the interfaces chemistry of both oxidized films was performed and is presented in the supplementary material (Figures S11, S12, S13). It should be, however, emphasized that the formation of silicides at Cu/Si(100) interfaces did not influence the formation and properties of the nanowires significantly.

## Discussion

### *In-situ* observations

The actual growing process has been monitored by the GISAXS measurements. The large footprint of the incidence beam on the investigated copper surface leads to statistically reliable results at short measurement times, making this technique a good candidate for *in-situ* characterization. On the other hand, the obtained results are integral parameters averaging the individual characteristics of a larger surface area. One must also take into account that the scattering results are in reciprocal space, which requires a real space model for interpretation.

Taking the incidence angle of 0.25° and the beam diameter of about 300 µm this footprint would be about 7 cm long. This is much longer than the size of specimen, which had an extension of only about 1 cm. Consequently the collected GISAXS data correspond to the average of a surface area of about 0.3 mm times 10 mm which is 3 mm². The critical angles of total reflection of Cu, Cu_2_O, and CuO are all within the range of 0.40° to 0.41°, which is larger than the incidence angle. Therefore, the incidence beam basically does not penetrate into the material except for the evanescent field of total reflection.

The scattering profiles shown in Fig. 3 have been computed only for cuts parallel to the surfaces, although two dimensional scattering patterns have been measured. This restriction was made because of the interference effects at the surface as described by the Distorted Wave Born Approximation “DWBA”^36,37^. These affect mainly the contributions normal to the surface and require an exact knowledge of the incidence angle for a correct description. Due to the thermal expansion of all the components within the sample cell during the heating experiment, it cannot be guaranteed that the incidence angle does not change slightly. This might change the aforementioned interferences and thereby inhibits the reliable use of the vertical components of the scattering patterns. Such a slight movement might change the intensity of the horizontal cuts, but in this case, it would be a simple multiplicative factor. The parameters used in this study, namely *L*_*c*_ and α, are both insensitive to such a change.

The restriction to horizontal cuts implies that only lateral changes in structure sizes can be observed, while the actual growth of nanowires is normal to the surface. The growth of nanowires, however, also leads to a signal in horizontal direction due to their cross section. A further restriction is caused by the accessible *q*-range. It corresponds to an accessible minimum size of 1.5 nm^38^, while the largest detectable sizes are on the order of 32 nm up to 80 nm depending on the minimum *q*-value. Smaller structures increase the constant background at large scattering angles. Structures that are larger than the upper size limit cannot be resolved, but lead to a power law behaviour at low *q*-values with a slope of 6-*D*, where *D* is the surface fractal dimension^39^. Since the diameter of the nanowires is more likely to be in the size range probed by GISAXS, the restriction to horizontal cuts should have a minor impact on the actual data interpretation.

The inaccessible low *q*-range, makes it also impossible to extrapolate the scattering curve unambiguously to *q* = 0. However, such an extrapolation would be needed in order to compute and interpret *L*_*c*_ truly as the correlation length as introduced by Porod. Therefore, an increase and decrease of the parameter *L*_*c*_ can only be treated as an indication for changing feature sizes at the surface, while its value cannot be directly linked to a typical structure size.

Despite of this lack of an absolute scale of this parameter, all three surfaces show some common features. The formation of the Cu_2_O layer does not influence *L*_*c*_, while a maximum is found at temperatures, where the CuO layer is formed. The increase of *L*_*c*_ can be explained by the formation of cupric oxide grains. Such grains have been reported to be on the size range of about 100 nm, which is close enough to the size window observable by GISAXS. The subsequent drop at higher temperatures is likely not to be an actual decrease in size but related to the GISAXS technique. Growing grains leave the window of sizes that are observable by this technique leading to a seemingly decrease in size.

The sputtered copper surface shows at higher temperatures the marked increase in *L*_*c*_. Growth of CuO nanowires explains this feature. The horizontal cuts in the GISAXS pattern mainly probe the cross section of the structures. They should be close or within the range that can be probed by small angle scattering making the nanowire formation directly visible. The actual growth in length is not probed due to the orientation of the cut, but the nanowires would anyhow outgrow the accessible size window soon.

The other two samples do not show this increase in *L*_*c*_ during the temperature change, but when they are being kept at a constant temperature of 450 °C. This behaviour points towards a kinetic effect, where nanowires grow most slowly on the surface deposited from vapour and faster on the foil. The sputtered surface favours obviously the fastest growth, since the formation that can be monitored by GISAXS has already been completed prior to reaching the constant temperature.

These observations on the parameter *L*_*c*_ can be correlated to the slopes of the scattering curves. The power law behaviour is related to the aforementioned dimensionality of the structures probed by small angle scattering. The actual values are difficult to interpret, since two components may affect them. First of all, the surface shows structures of different sizes, *e.g*. oxide grains. Such a polydispersity lead to a superposition of the individual scattering contributions. If the grains could be described by a fractal aggregate, the scattering curve would show a power law with slope –*d*, where *d* is the mass fractal dimension^40^. As stated above, a fractal surface leads to a power law slope of –(6-*D*), where *D* is the surface fractal dimension. Given the fact that different types of structures are present, the observed slope could be caused by a combination of both power law behaviours. A steeper slope may indicate a more compact structure in terms of mass fractal or a smoother surface in terms of surface fractal. Nevertheless, both possible changes indicate a loss of interstices either between the structures or at the surface and thereby a more compact structure.

The steepening of the slope of the scattering profiles measured at the sputtered surface above 300 °C and at the other surfaces, when kept constant at 450 °C, coincides with the increase observed in *L*_*c*_. It has been reported that CuO nanowires are single crystals or twinned crystals^23,41,42^. Consequently their surfaces are likely to be smooth. A smooth surface has a dimensionality of 2, while the dimensionality of fractal surfaces is higher. One should therefore expect that the formation of smooth surfaces should result in a power law exponent that approaches –(6-2) = −4. This value is not actually observed for these scattering curves, but the trend towards steeper slopes points in this direction.

The copper foil and the sputtered copper surface show a dense layer of long nanowires. This is in good agreement with the strong change observed for the GISAXS parameters of these two surfaces when heated. The loose layer of short nanowires found on the vapour deposited surface should lead to a weak change of the GISAXS signal, which is actually the case. However, even these few nanowires have a diameter that is at least on the same order of magnitude, like in the other cases. Therefore, one might ask, why the parameter *L*_*c*_, which is related to the average size of the structures, increases only by such a small amount. The fact that one obtains a finite value even without the presence of nanowires at lower temperatures proves that other structures like the grains at the surface influence the result. Consequently, the value of *L*_*c*_ is an average parameter influenced by grain sizes and nanowire sizes. Growing nanowires just shifts the weight of the average more towards the value corresponding to their cross section.

Growth conditions have been identical for all three samples. Therefore, the observed differences in nanowire growth are caused by the metallic copper underneath the growing oxide layers. Possible factors are the substrate, sample composition, surface roughness, grain size, and grain orientation.

### Sputtered copper film

The grains of the sputtered film are small (Fig. 2c). Their diameter is similar or smaller compared to the grain sizes reported for the Cu_2_O layer on copper foil during thermal oxidation experiments^24^. Moreover, the nucleating islands on Cu(111) tend to align and coalescent^43^, which may be related to the ordered “29” lattice structure of chemisorbed oxygen on such surfaces^44^. Consequently, one can assume that each copper grain of the sputtered surface nucleates one grain of the Cu_2_O layer, which is small compared to the usual size of such grains. Therefore, the surface of the cuprous oxide grains is large leading to a relative high flux of material by means of grain boundary diffusion. Similarly, the fine Cu_2_O grains result in fine CuO grains. Since copper oxide nanowires grow from the top edge of the cupric oxide grains^26^, these fine grains result in a high number of possible nanowires. Consequently, one expects a high density of copper nanowires on this surface. Such a relation between small grains of the copper substrate and strong growth of copper nanowires has also been observed by Liang and co-workers^45^.

The flux of copper from the metal-cuprous oxide interface to the nanowire is essential for nanowire growth^26^, and depends on the grain boundary diffusion through the two oxide layers. Therefore it is a function of the diffusion coefficient and of the fraction of the area that is covered by grain boundary layers and increases with greater area fraction and with higher temperatures. The small diameters of the Cu_2_O grains on the sputtered surface lead to a large area fraction occupied by grain boundaries. This can compensate for smaller diffusion coefficients at lower temperatures and lead to the same flux as through films containing of larger grains at higher temperatures. Therefore, one expects sufficient material diffusion for the onset of nanowire growth even at relative low temperatures, like at the observed 350–400 °C, while the other two studied samples show such growth only when kept at 450 °C.

The XRD data also agree with a fast diffusion within the film in the case of the sputtered surface. The other two samples show after temperature treatment the signatures of the original copper and of both of the oxides. The pattern of the sputtered film, however, has basically no sign of a lower oxidation state of copper than CuO. This indicates that diffusion within the film was fast enough for metallic copper and for cuprous oxide to react completely to cupric oxide within the time frame of the experiment.

### Copper foil

The grains of the copper foil are large and one has to assume that more than one island of cuprous oxide nucleates and grows on each. In addition, the surface is very rough which has been reported to enhance the growth of smaller grains of cuprous as well as cupric oxide. This consequently leads to enhanced CuO nanowire growth^28^ and is caused by the inhibition of the free diffusion of oxygen, which reduces the probability that an oxygen atom is captured by an already nucleated oxide island. Therefore, a considerable large fraction of the surface should be covered by grain boundaries enabling grain boundary diffusion and consequently to nanowire growth. The growth of nanowires has been observed in GISAXS at a higher temperature compared to the sputtered surface. This is in agreement with a reduced flux and corresponds to the lower nanowire density found in the SEM images.

The roughness may also explain another observation, namely the relatively high fluctuation in nanowire density when comparing different areas of size 1 µm², which is 16% in the case of the sputtered surface, but 41% in the case of the copper foil. Based on the topography of the foil’s surface (Fig. 1a) oxygen diffusion on the surface may be inhibited locally very differently by surface features. However, the orientation of the grains may also play a role, since different facets of copper crystals are exposed at the surface of the foil. The nucleation density of cuprous oxide islands during the initial phase of oxidation depends on the copper facet^43,46^ with a higher nucleation density on Cu(110) than on Cu(100). The data present do not allow for a discrimination of these two effects.

The copper foil’s surface after thermal oxidation lacks, according to the SEM image (Fig. 6a), nanowires within a valley, where other, globular structures have grown. This observation may be explained by the stresses at the copper surface. Compressive stress reduces the growth of nanowires^24,27^. Given the densities of copper^47^, cuprous oxide^48^, and cupric oxide^11^ it is obvious that the molar volume copper of 7.09 cm³/mol is considerably smaller than that of cupric oxide with 12.11 cm³/mol, while the volume occupied by cuprous oxide (CuO_0.5_) is within the range of 11.65 cm^3^/mol to 12.44 cm³/mol and thereby close to the one of cupric oxide. Therefore, especially the oxidation of copper to Cu_2_O leads to an increase in volume. The confined space within a valley restricts the growth of the oxide layer leading to compressive stresses in the oxide layer. This compression is likely to suppress in consequence the growth of nanowires within the valley.

### Vapour deposited copper film

Evaporated copper samples reveal the least of nanowire growth in terms of number density. Compared to sputtered Cu and foil, the growth is much slower, as seen in the late increase of *L*_*c*_ in the GISAXS data (Fig. 4) and the short wires in the SEM image (Fig. 6b). This is in agreement with the other samples, where high density was linked to fast growth. The surface of evaporated copper combines grains that are much larger than in the case of the sputtered one with the smallest roughness of all studied samples. Both effects do not inhibit the growth of the Cu_2_O grains and consequently one expects large grains and a small fraction of the area covered by grain boundaries of cuprous oxide. Therefore the flux of copper ions should be relatively small leading to a weak growth of nanowires. A second factor that could play a role is the influence of the substrate. El Mel and co-workers^49^ found a strong increase in nanowire growth for film thicknesses from 300 nm to 700 nm. Similarly Wang and co-workers^50^ have reported that no nanowires grew on a film of thickness 0.5 µm on a Ti/Si substrate, while growth was observed on a similar film of 1 µm thickness. This is likely to be caused by stresses since Chen and co-workers have observed nanowire growth at the edge of a patterned film of 400 nm thickness^27^. The thermal expansion of copper (16.5·10^−6^ K^−1 ^^51^) is considerably larger than that of the silicon substrate (2.6·10^−6^ K^−1 ^^52^) and of the titanium layer (8.6·10^−6^ K^−1 ^^51^). Heating the sample consequently induces compressive stresses at the bottom of the copper film. Such stresses are reduced more easily within a film of 750 nm thickness, like in the case of the sputtered surface, than within 550 nm, like in the case of the vapour deposited film. As stated above, compressive stress inhibits nanowire growth, which might explain the reduced growth on this surface.

## Conclusions

The growth of CuO nanowires was investigated on three different Cu surfaces. All three types showed a different formation temperature, thickness, lengths morphology and number density. The growth of CuO nanowires on different substrates can be explained well by means of grain boundary diffusion of copper. A high number of grain boundaries promotes flux as can be seen on the sputtered copper surface and on the copper foil. Roughness prevents the formation of larger grains of CuO on the Cu surface and enhances therefore the density of grain boundaries.

Stresses at the copper-cuprous oxide boundary also influences the growth. The compressive stresses in the thin film prepared by vapour deposition and within a valley of the copper foil reduce or inhibit nanowire growth. The foil sample exhibits the largest grain size of Cu, but also the highest roughness triggering the growth of fine Cu_2_O grains. This leads to the formation of many long NW.

The sputtered copper surface, which has a fine grain structure and a significant roughness exhibits the highest number density of nanowires and has also growth at the lowest temperatures. The film deposited from evaporated copper, having comparable larger grain size and a lower roughness on the other hand, has the lowest number density of nanowires, and these nanowires grow most slowly.

## Supplementary information

Supporting Information - Study of CuO Nanowire Growth on Different Copper Surfaces

## References

[1] Kasinathan K, Kennedy J, Elayaperumal M, Henini M, Malik M (2016). Photodegradation of organic pollutants RhB dye using UV simulated sunlight on ceria based TiO_2_ nanomaterials for antibacterial applications. Sci. Rep..

[2] Fang F (2011). Size-controlled synthesis and gas sensing application of tungsten oxide nanostructures produced by arc discharge. Nanotech..

[3] Kennedy J (2017). Synthesis and enhanced field emission of zinc oxide incorporated carbon nanotubes. Diamond Related Mater..

[4] Fang F, Kennedy J, Carder D, Futter J, Rubanov S (2014). Investigations of near infrared reflective behaviour of TiO_2_ nanopowders synthesized by arc discharge. Opt. Mater..

[5] Magdalane CM (2017). Evaluation on the heterostructured CeO_2_/Y_2_O_3_ binary metal oxide nanocomposites for UV/Vis light induced photocatalytic degradation of Rhodamine - B dye for textile engineering application. J. Alloys Compd..

[6] Santra K, Sarkar CK, Mukherjee MK, Ghosh B (1992). Copper oxide thin films grown by plasma evaporation method. Thin Solid Films.

[7] Sambandam A, Wen X, Yang S (2005). Room temperature growth of CuO nanorod arrays on copper and their application as a cathode in dye-sensitized solar cells. Mater. Chem. Phys..

[8] Feng Y, Zheng X (2010). Plasma-Enhanced Catalytic CuO Nanowires for CO Oxidation. Nano Lett..

[9] Li D, Hu J, Wu R, Lu JG (2010). Conductometric chemical sensor based on individual CuO nanowires. Nanotech..

[10] Liao L (2009). Multifunctional CuO nanowire devices: p-type field effect transistors and CO gas sensors. Nanotech..

[11] Lupan O (2016). Single and networked CuO nanowires for highly sensitive p-type semiconductor gas sensor applications. physica status solidi (RRL) – Rap. Res. Lett..

[12] Steinhauer S (2013). Gas sensing properties of novel CuO nanowire devices. Sens. Actuators, B.

[13] Filipič G, Cvelbar U (2012). Copper oxide nanowires: a review of growth. Nanotech..

[14] Pfefferkorn G (1953). Elektronenmikroskopische Untersuchungen über den Oxydationsvorgang von Metallen. Naturwiss..

[15] Gulbransen, E. A., Copan, T. P. & Andrew, K. F. Oxidation of Copper between 250 °C and 450 °C and the Growth of CuO “Whiskers”. *J. Electrochem. Soc*. **108**, (1961).

[16] Jiang X, Herricks T, Xia Y (2002). CuO Nanowires Can Be Synthesized by Heating Copper Substrates in Air. Nano Lett..

[17] Huang LS (2004). Preparation of large-scale cupric oxide nanowires by thermal evaporation method. J. Cryst. Growth.

[18] Xu CH, Woo CH, Shi SQ (2004). Formation of CuO nanowires on Cu foil. Chem. Phys. Lett..

[19] Kumar A, Srivastava A, Tiwari P, Nandedkar V (2004). R. The Effect of Growth Parameters on the Aspect Ratio and Number Density of CuO Nanorods. J. Phys.: Condens. Matter.

[20] Proust JL (1799). Recherches sur le Cuivre. A. Chem.

[21] Wieder H, Czanderna AW (1962). The Oxidation of Copper Films to CuO_0.67_. J. Phys. Chem..

[22] Mimura K, Jae Won L, Isshiki M, Zhu Y, Jiang Q (2006). Brief review of oxidation kinetics of copper at 350 °C to 1050 °C. Metallurg. Mater. Trans. A-Phys. Metallur. Mater. Sci..

[23] Liang J, Kishi N, Soga T, Jimbo T (2010). Cross-sectional characterization of cupric oxide nanowires grown by thermal oxidation of copper foils. Appl. Surf. Sci..

[24] Mema R, Yuan L, Du Q, Wang Y, Zhou G (2011). Effect of surface stresses on CuO nanowire growth in the thermal oxidation of copper. Chem. Phys. Lett..

[25] Gonçalves AMB, Campos LC, Ferlauto AS, Lacerda RG (2009). On the growth and electrical characterization of CuO nanowires by thermal oxidation. J. Appl. Phys..

[26] Yuan L, Wang Y, Mema R, Zhou G (2011). Driving force and growth mechanism for spontaneous oxide nanowire formation during the thermal oxidation of metals. Acta Mater..

[27] Chen M, Y ue Y, Ju Y (2012). Growth of metal and metal oxide nanowires driven by the stress-induced migration. J. Appl. Phys..

[28] Yuan L, Zhou G (2012). Enhanced CuO Nanowire Formation by Thermal Oxidation of Roughened Copper. J. Electrochem. Soc..

[29] Braud F, Torres J, Palleau J, Mermet JL, Mouche MJ (1995). Ti-diffusion barrier in Cu-based metallization. Appl. Surf. Sci..

[30] Fritz-Popovski G, Bodner SC, Sosada-Ludwikowska F, Maier G (2018). A new device for high-temperature *in situ* GISAXS measurements. Rev. Sci. Instrum..

[31] Huang TC, Toraya H, Blanton TN, Wu Y (1993). X-ray powder diffraction analysis of silver behenate, a possible low-angle diffraction standard. J. Appl. Crystallogr..

[32] Porod G (1951). Die Röuml;ntgenkleinwinkelstreuung von dichtgepackten kolloidalen Systemen. I. Teil. Kolloid-Z..

[33] Weber G, Gillot B, Barret P (1983). Interfaces structure in relation with the mechanisms in the reaction copper-silicon. Physica Status Solidi (A).

[34] Myers SM, Follstaedt DM (1996). Interaction of copper with cavities in silicon. J. Appl. Phys..

[35] He Y, Brown C, Lundgren CA, Zhao Y (2012). The growth of CuSi composite nanorod arrays by oblique angle co-deposition, and their structural, electrical and optical properties. Nanotech..

[36] Sinha SK, Sirota EB, Garoff S, Stanley HB (1988). X-ray and neutron scattering from rough surfaces. Phys. Rev. B.

[37] Rauscher M, Salditt T, Spohn H (1995). Small-angle x-ray scattering under grazing incidence: The cross section in the distorted-wave Born approximation. Phys. Rev. B.

[38] Glatter, O. In *Small Angle X-ray Scattering*, Glatter, O. and Kratky, C., editors, chapter Data Treatment. Academic Press, London (1982).

[39] Bale HD, Schmidt PW (1984). Small-Angle X-Ray-Scattering Investigation of Submicroscopic Porosity with Fractal Properties. Phys. Rev. Lett..

[40] Teixeira J (1988). Small-Angle Scattering by Fractal Systems. J. Appl. Cryst..

[41] Kaur M (2006). Growth and branching of CuO nanowires by thermal oxidation of copper. J. Cryst. Growth.

[42] Sheng H (2016). Twin structures in CuO nanowires. J. Appl. Cryst..

[43] Zhou G, C. Yang J (2005). Initial Oxidation Kinetics of Cu(100), (110), and (111) Thin Films Investigated by *in Situ* Ultra-high-vacuum Transmission Electron Microscopy. J. Mater. Res..

[44] Matsumoto T (2001). Scanning tunneling microscopy studies of oxygen adsorption on Cu(1 1 1). Surf. Sci..

[45] Liang J, Kishi N, Soga T, Jimbo T (2011). The Synthesis of Highly Aligned Cupric Oxide Nanowires by Heating Copper Foil. J. Nanomater..

[46] Luo L, Kang Y, Yang JC, Zhou G (2012). Effect of oxygen gas pressure on orientations of Cu_2_O nuclei during the initial oxidation of Cu(100), (110) and (111). Surf. Sci..

[47] Eckerlin, P. & Kandler, H. volume 6, chapter Structure Data of Elements and Intermetallic Phases. Ac-Mn. Springer-Verlag Berlin Heidelberg (1971).

[48] In *Landolt-Börnstein - Group III Condensed Matter*, Madelung, O., Rössler, U. & Schulz, M., editors, volume 41 C. Springer-Verlag Berlin Heidelberg (1998).

[49] el Mel A-A (2013). Growth control, structure, chemical state, and photoresponse of CuO-CdS core-shell heterostructure nanowires. Nanotech..

[50] Wang Y (2011). Formation of CuO nanowires by thermal annealing copper film deposited on Ti/Si substrate. Appl. Surf. Sci..

[51] In *CRC Handbook of Chemistry and Physics*, Lide, D. R., editor. Taylor & Francis Group Boca Baton, Florida88^*th*^ edition (2008).

[52] Watanabe H, Yamada N, Okaji M (2004). Linear Thermal Expansion Coefficient of Silicon from 293 to 1000 K. Int. J. Thermophys..

