# Supplementary material for: Study of CuO Nanowire Growth on Different Copper Surfaces
Source: Sci Rep. 2019 Jan 28;9:807. doi: 10.1038/s41598-018-37172-8 (PMC6349934; doi:10.1038/s41598-018-37172-8)
Supplement: Supplementary file 1 — Supporting Information - Study of CuO Nanowire Growth on Different Copper Surfaces [file 41598_2018_37172_MOESM1_ESM.docx]

Supporting Information

Study of CuO Nanowire Growth on Different Copper Surfaces

G. Fritz-Popovski^1,2^, F. Sosada-Ludwikowska^1^, Anton Köck^1^, Jozef Keckes^3^ and G. A. Maier^1^

^1^Material Center Leoben GmbH, ^2^ Institute of Physics, ^3^ Department Materialphysics, Montanuniversität Leoben, all: 8700 Leoben, Austria

# Surface Roughness

The surface roughness was determined using AFM analysis in tapping mode, scan mode AC. The damping setpoint was set to 50% and a frequency of 245 kHz was used. The size of the scans were 3x3µm² with a resolution of 256x256 points. Figure S1 is an extension to Figure 1 of the paper. From 2D images and the corresponding linecuts the difference between the samples in terms of frequency of roughness and height variations can clearly be seen.


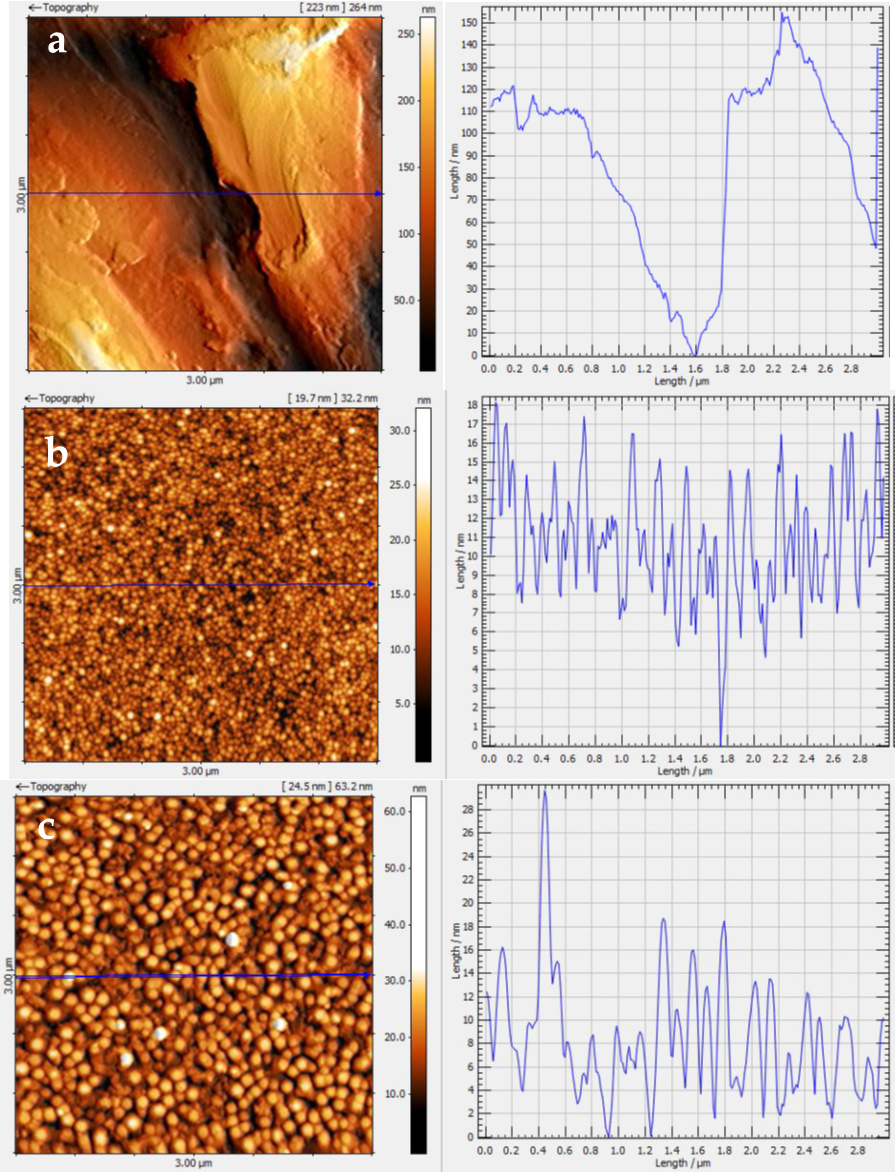


**Figure S1**: Atomic force microscopy in 2D and corresponding linecus of the initial (a) copper foil, (b) evaporated copper, and (c) sputtered copper. The areas shown are 3×3 µm2 large.

The results of roughness analysis using Scantool software (Semilab DME) are summarized in Table S1.

|  |  |  |  |
| --- | --- | --- | --- |
|  |  | | |


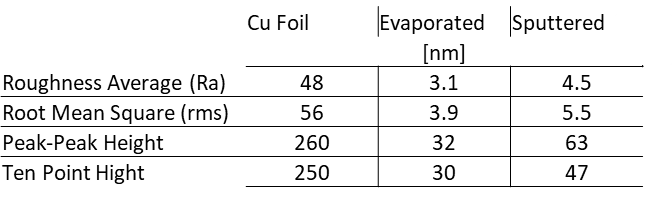


Table S1: Results from AFM analysis for all three samples. For the discussion in the paper the Ra values were used.

# Grain Size Distribution

The grain sizes were determined using electron back scattering diffraction. The corresponding size distributions are plotted in Figure S2.

**Figure S2**: Grain sizes at the surface of copper foil (red), evaporation deposited copper (blue) and copper deposited by sputtering (black).

These distributions correspond to average grain diameters of 4.2±3.4 nm for the foil, 1.12±0.65 nm for the surface prepared by evaporation of copper and 0.26±0.13 nm for the sputter deposited copper. Especially the data from the copper foil are prone to show considerable uncertainties caused by the high surface roughness, which impeded exact measurements.

# Inverse Pole Figure Maps Overlaid with Grayscale Image

The inverse pole figure maps of the surfaces show the discussed grain sizes and orientations. These features are partly connected to the height profile of the surface. This can be seen, when they are overlaid with the grayscale images obtained by scanning electron microscopy and compared to Figure 2 of the paper.

The map obtained from the copper foil (Figure S3a) shows that there is some alignment of the grains with the parallel valleys seen in the microscopic image. The large surface roughness makes it difficult to access the signal from several areas of the image.

The map from measured surface of evaporated copper (Figure S3b) shows a clear relation the grey-scale shading with the grains. While most of the grains exhibit the (111) surface, some of the grains show different surfaces.

In the case of copper deposited by sputtering (Figure S3c), however, there is a correlation of the surfaces that are not oriented in (111) direction and the grains. Basically all these surfaces are clearly correlated to the interstices between the islands, while the (111) surfaces are mostly shown on the islands.

# Background of GISAXS Fits

The GISAXS data have been approximated by a power low combined with a constant background. The resulting background data are shown in Figure S4. This parameter is sensitive to the amount of structures that are smaller than the size range covered by the *q*-range of the experiment. This corresponds in this case according to Fourier transformation theory to a maximum size of about π/*q*_max_≈1.5 nm.

One should keep in mind that multiplying the scattering curve with a constant factor will change the background by the same factor. A slight change of the incidence angle during the heating experiment and the connected thermal expansion of all the materials within the cell might cause such an effect. Despite of the fact that the design of the heating chamber should minimize such movements, they cannot be ruled out inhibiting a detailed interpretation of the background data.

The background only changes slightly up to about 375°C in the case of the copper foil. Thereafter, it decreases. This trend is continued when observed at constant 450°C. Since this is the regime, where strong growth of nanowires has been observed it might reflect their smooth crystalline surfaces.

**Figure S4**: Background obtained from the power law model approximated to the GISAXS profiles of data measured (a) during heating and (b) at 450°C. Red: copper foil, blue: deposited from evaporated copper, and black: from sputtered copper. Temperatures of oxidation to cuprous and to cupric oxide are shaded.

**Figure S3** Inverse pole figure maps overlaid with grey scale electron microscopy images of (a) copper foil, surfaces prepared by (b) deposition of evaporated, and (c) sputtered copper.

The background of the surface deposited from evaporated copper does not show such a clear trend. There is however a slight increase at the upper limits of both temperature ranges, where a new oxide is formed. There is a slight trend to lower background values at high temperatures and at constant 450°C, which might also reflect the increase in flat crystalline surfaces of the nanowires.

Please note that the sample with the sputtered copper film developed due to thermal movements a reflection from one of the substrate edges at 300°C. It had to be shifted laterally by 300µm in order to remove this reflection. Consequently it is not appropriate to compare intensities values and backgrounds after this movement to the ones prior to it, due to this different measured position on the surface.

The background of the GISAXS data of the sputtered copper surface also increases within both of the temperature ranges, where new oxides are formed. Contrary to the other two surfaces, the background data at temperatures above 300°C are closely related to the slope (see Figure S6), *i.e.* a steep slope corresponds to a high background. This indicates some numeric instability of the least squares problem. One should refrain from interpreting the background data in this case, since the determined slope is more stable than the background.

# 2D GISAXS patterns

The GISAXS measurements result in 2D scattering patterns (Figure S5). The evaluation was based solely on the horizontal cuts that contain the information on changes in lateral structure size or arrangement. Basically, one could evaluated the two dimensional whole information of the scattering patterns. This would require, as discussed, detailed information on the reflections from the surface, which are highly sensitive to changes in incidence angle and therefore only possible with great efforts for these samples.

The measured patterns indicate that the main changes observed are within the evaluated horizontal cuts, while the vertical components do hardly change at all. This can be explained by two factors: First of all the pattern shows a central streak, which is caused by sample imperfections. Changes of scattering signal are weak compared to this high background. Additionally, the main change in terms of nanowire growth would be the increasing length of the structures. Given the relatively great length of the nanowires, they soon outgrow the size range that can be observed by GISAXS in vertical direction. Consequently a detailed evaluation of the whole 2D pattern would result in little additional information on the system.

**Figure S5**: GISAXS patterns of copper foil (a-c), of a surface deposited from evaporated copper (d-f) and of a sputtered copper surface (g-i). Patterns (a), (d), and (g) have been collected at 25°C, (b), (e), and (h) upon reaching 450°C and (c) (f), and (i) after 2 h at 450°C.

# Fast Thermal Treatments

Growth of nanowires and the grain boundary diffusion of copper are both time dependent phenomena. Therefore, the time dependence during the heating experiment is of interest.

The fast ramping experiments with heating rates of 10°C required also fast collection of data. The scattering curves and the parameters obtained from them have considerably higher statistical uncertainties than the ones collected in the step wise mode.

The parameters, however, show good agreement with the ones collected in the step-wise mode (Figure S6). There are three differences of importance:

First of all, the foil shows a remarkably different behaviour of *L_c_* for the two heating experiments. Once, structures are seen just when the Cu_2_O grains have formed, once, when CuO grains have formed. It is not completely sure, why this is the case. However, both these peaks in *L_c_* are linked to temperatures just above the temperature range, where new oxides form. When the formation of nanowires can be expected, *i.e.* at temperatures above 350°C, the signals of both experiments are basically identical.

**Figure S6**: Parameters *L_c_* (a, c, e) and α (b, d, f) as determined by step-wise temperature changes (solid circles and lines), and by ramping (open circles and dashed lines) obtained from GISAXS measured at a foil (a, b), at a surface deposition from evaporated copper (c, d), and at a surface prepared by sputtering copper (e, f).


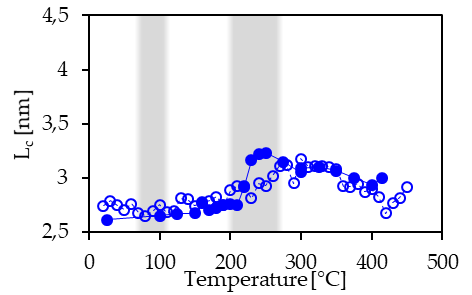

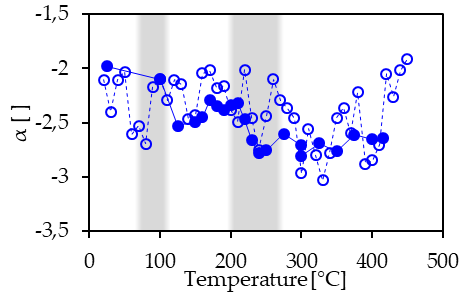

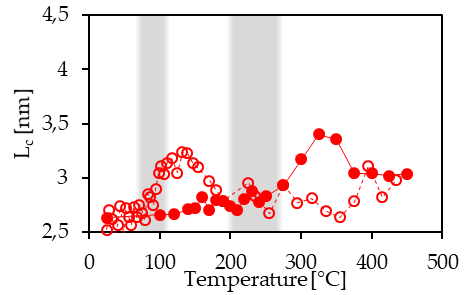

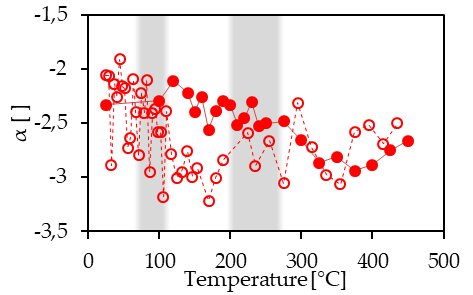

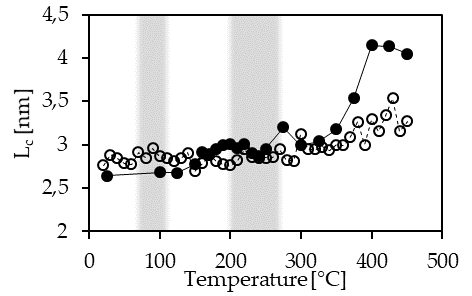

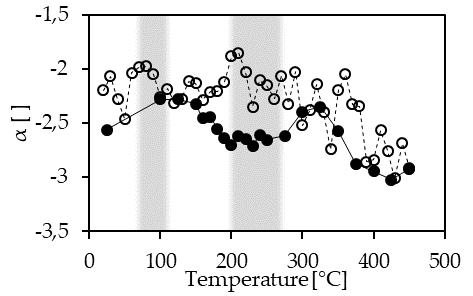


a

b

c

d

e

f

Secondly, the maximum in *L_c_* of the surface prepared by deposition of vapour is shifted slightly to higher temperatures. Most probably this signal corresponds to the growth of CuO grains. This process takes some time and has not been finished, when the next data point at higher temperature has been measured.

Thirdly, the growth of nanowires on the sputtered surface is less pronounced in the ramping experiment than in the step-wise measurements. This is most likely explained by the finite flux that does not allow enough copper atoms to diffuse to the nanowires within the short times available. Despite of this, the increase of *L_c_* with temperature is strongest for this sample during rapid heating.

# Vertical Cuts through Scattering Patterns

Vertical cuts through the GISAXS scattering patterns (Figure S7) are prone to uncertainties. Nevertheless, parameters derived from such profiles may be used in order to compare results with those of the horizontal cuts. The data presented here have not been computed from a central cut, which would have the additional feature of a specular reflection peak. Two cuts that were positioned 0.14 nm^-1^ at the left and at the right hand side of the primary beam were computed.

**Figure S7**: Vertical GISAXS profiles of data measured (a,c,e) during heating and (b,d,f) at 450°C. (a,b ) copper foil, (c,d) deposited from evaporated copper, and (e,f) from sputtered copper. Curves are coloured from blue to red with increasing temperature or time. The curves shown here have been obtained left of the primary beam.

The profiles cannot be described by a simple exponential decay due to the reflection and interference effects, wherefore the slope and parameter α cannot be determined. The parameter *L_c_*, however, (Figure S8) shows a behaviour that is similar to the one observed for the horizontal cuts at least for two different the films on a substrate. On the other hand the copper foil shows a pronounced increase of *L_c_* with temperature at about 300°C. This corresponds to the increase found in the horizontal cuts, but here, no drop at higher temperatures is observed.

If we compare the development of *L_c_* with time at 450°C, we observe an increase similar to the one seen for the horizontal cuts. The result from the vertical cuts, however, shows two pronounced steps after 40 minutes and after 80 minutes. These steps are most likely due to the movements and the corresponding shifts of the interferences with reflected beams. Therefore, the height of these steps of about 1 nm gives allows for estimating for the uncertainty due to such movements. Such movements can also happen during the heating phase of the sample. Consequently, the actual change of the parameter *L_c_* us highly uncertain for vertical cuts and should not be interpreted.

Please note that the two steps seen in Figure S7b correspond to the times, when the constant background obtained by approximating an exponential decay to the horizontal cuts (Figure S4b). Both parameters, *L_c_* in the vertical cuts and *b* in the horizontal cuts, depend on the interference effects of direct beams and beams reflected at the surface. Therefore, both of them are sensitive to the incidence angle of the primary beam. One might therefore conclude that the copper foil moved at these times within the sample chamber.

**Figure S7**: Parameter *L_c_* obtained from vertical GISAXS profiles of data measured (a) during heating and (b) at 450°C. Red: copper foil, blue: deposited from evaporated copper, and black: from sputtered copper. Temperatures of oxidation to cuprous and to cupric oxide are shaded. Points marked with solid symbols have been measured left of the primary beam, open points at the right hand side.

# Distribution of facets on original surfaces

The inverse pole figures of the copper surfaces (Figure S8) show that the copper foil has no preferred orientation. The film deposited from vapour has the discussed preferred {111} surfaces with some contribution of {001} surfaces. The sputtered film is strongly favouring the {111} surfaces. Other surfaces also exist mainly at the interstices of the surface features (Figure S3). Since they also show some preferred orientations, one might assume them to be facets of the crystallites growing in [111] direction.

**Figure S8**: Inverse pole figures of (a) copper foil, (b) copper deposited after evaporation and (c) on a sputtered copper film.

# XRD peak not corresponding to Cu, Cu_2_O or CuO

A peak was observed in the XRD pattern of the sputtered sample after heat treatment close to 2ϑ=44.6°. Its position does not correspond to the patterns of the initial elements Cu, Si, and Ti, nor to their oxides, but to Cu_3_Si. The only other material that might form and have a peak nearby is rutile (Figure S9). However, the most prominent peak of rutile would be expected at 27.4°, while the peak at 44.0° is weak. Contrary to this Cu_3_Si has its dominant peak at 44.8°, while other peaks are much weaker.

# Estimation of strains

Strain influences the position of peaks measured by XRD. Relating the peak position relative to the position of the unstrained material can therefore result in information on strains within the individual layers of the sample.

Such an evaluation is only possible, if enough scattered intensity has been detected for a phase within the sample. Figure S10a demonstrates that metallic copper can be clearly detected within the copper foil as well as within the vapour deposited film. The same is true for Cu_2_O. The sputtered sample shows basically only peaks that can be explained by CuO as well as the one Cu_3_Si peak, while Cu and Cu_2_O are absent. Therefore, it should be possible to obtain reliable strain data for CuO in all samples, while Cu and Cu_2_O peak positions cannot be determined reliably for the sputtered copper film.

**Figure S9**: Peak positions of possible components of the sputtered surface after heat treatment.

**Figure S10**: (a) Fraction of XRD peaks that can be attributed to a phase relative to total area under curve. (b) Strain of phases estimated from XRD peak positions.

Figure S10b shows the resulting strain data based on the following crystallographic lattice data:

- Copper^[[1]](#endnote-1)^: $Fm\bar{3}m$, *a* = 3.62540 Å
- Cuprous oxide^[[2]](#endnote-2)^: $Pn\bar{3}m$, *a* = 4.2696 Å
- Cupric oxide^[[3]](#endnote-3)^: $C2/c$, *a* = 4.6881 Å, *b* = 3.4228 Å, *c* = 5.1321 Å, β = 99.497°.
- Cu_3_Si: hexagonal, *a* = 4.04 Å, *c* = 2.44 Å.

The position of the Cu_3_Si peak agrees with the lattice parameter given in literature, but these literature values are not precise enough for a detailed evaluation of the strain within this phase. Consequently, only strain values of CuO have been determined for the sputtered sample.

The metallic copper shows for the copper foil and for the vapour deposited film signs of compression. The cuprous oxide shows considerable expansion in the case of the foil but basically no deformation in the vapour deposited film. The CuO lattice is not deformed in all three films.

# Structure of Cu-Films by FIB cross sectioning

The structure of the films was investigated using FIB and EDX. Key parameter under investigation was the formation of Cu_3_Si and a qualitative check if this evaluation is valid. As deduced from SEM cross sections and the corresponding EDX spectra, shown in Figure S11, oxygen can be found between 2 and 2.5µm inside the Cu foil. In the sputtered and the evaporated Cu samples the whole film was oxidized. The Cu-Si precipitates at the interface in the vapour deposited Cu were clearly visible (Figure S11,b). In addition, incooperated Si was found everywhere in the sputtered and evaporated samples. This indicates, that the diffusion barrier of SiO2 on the wafer was not sufficient to separate the Si from the Cu and vice-versa. Detailed analysis of the chemical composition of shown in term of line profiles in Figure S12 and in more precise area averages in Figure S13.


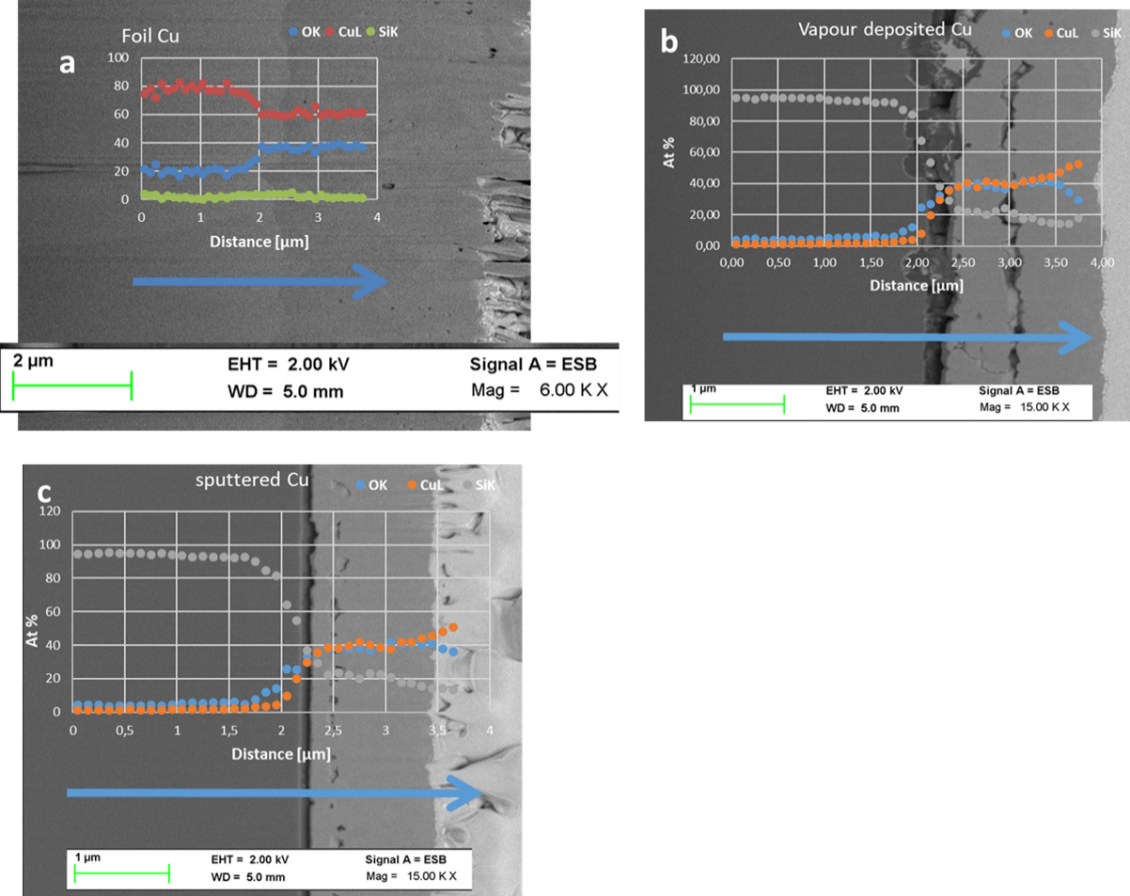


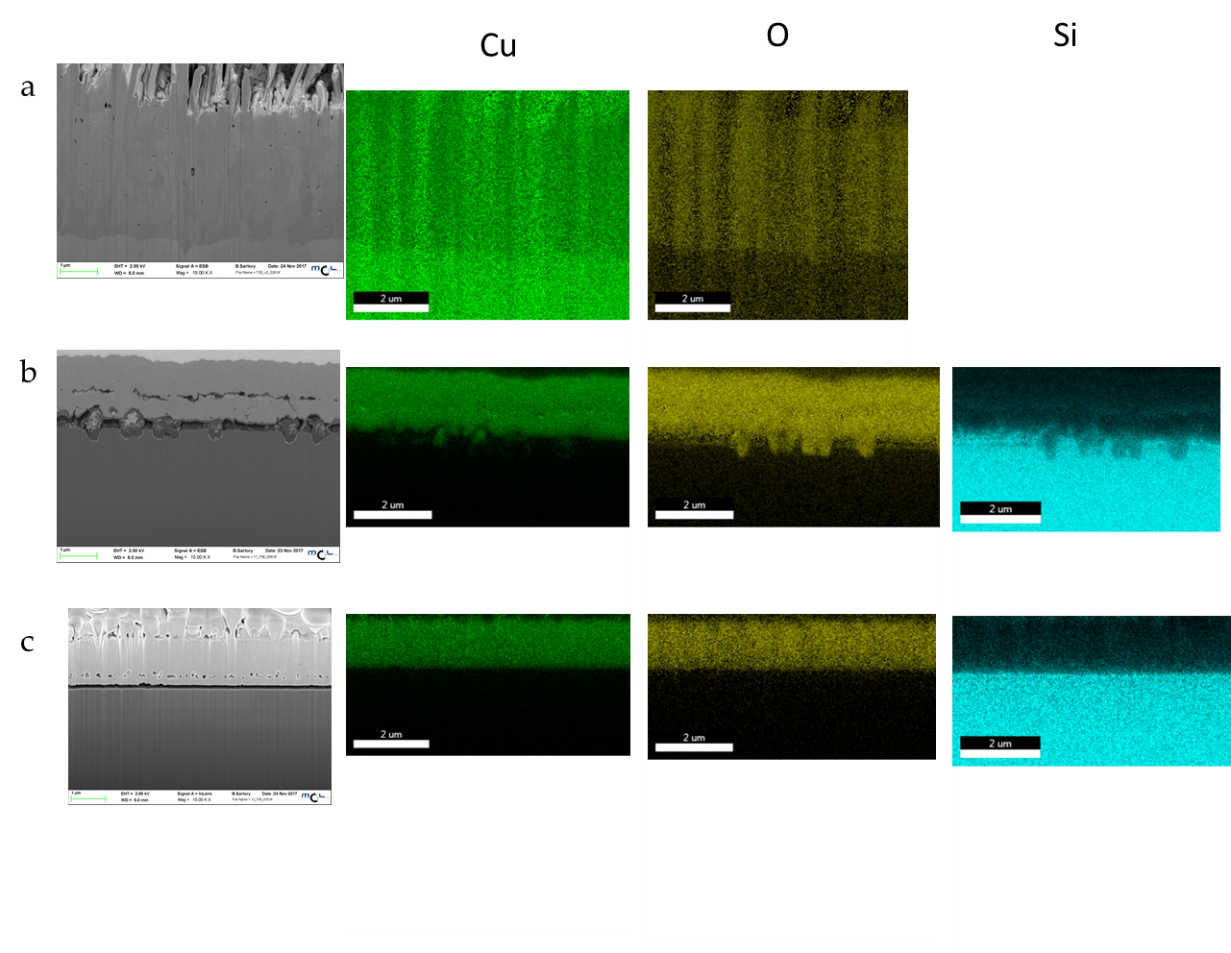


**Figure S11:** Images and EDX colour maps from FIB cross sections of the oxidized Cu samples: (a) copper foil, (b) vapour deposited copper, and (c) sputtered copper.

**Figure S12:** Chemical composition as determined by EDS on FIB cross sections and corresponding line profiles: (a) copper foil, (b) vapour deposited copper, and (c) sputtered copper.


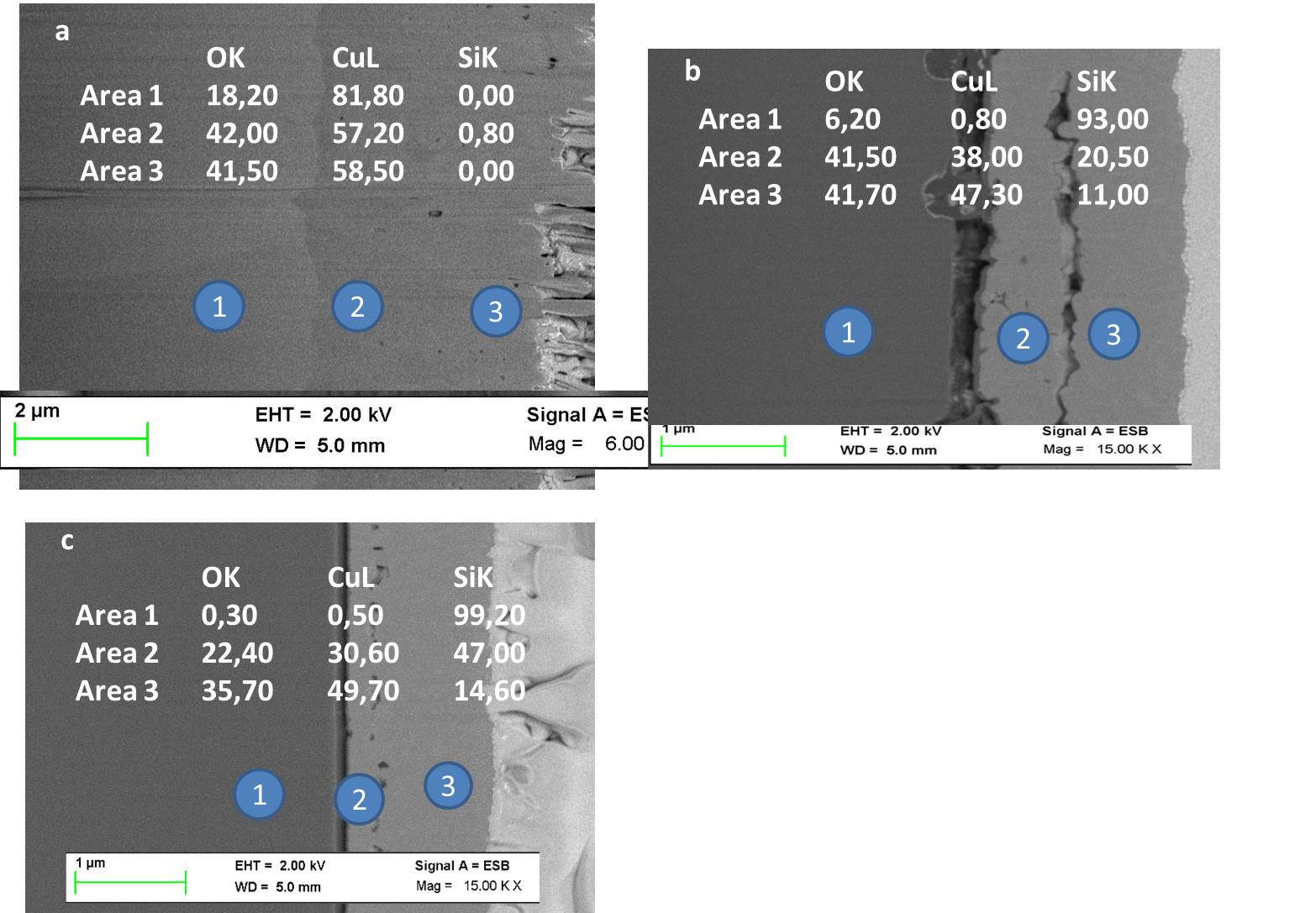


**Figure S13:** Chemical composition as determined by EDS on FIB cross sections and corresponding area mean values: (a) copper foil, (b) vapour deposited copper, and (c) sputtered copper.

1. Robert M.C., Saravanan R., Saravanakumar K., Rani M.P.: Structural analysis of Al, Ni and Cu using the maximum entropy method, multipole and pair distribution function. *Z. Naturf. A* **64** (2009) 361-369 [↑](#endnote-ref-1)
2. Kirfel A., Eichhorn K.D.: Accurate Structure Analysis with Synchrotron Radiation. The Electron Density in Al_2_O_3_ and Cu_2_O. *A. Cryst. A* **46** (1990) 271-284 [↑](#endnote-ref-2)
3. Langford J.I., Louer D.: High-resolution powder diffraction studies of copper(II) oxide*. J Appl. Cryst.* **24** (1991) 149-155 [↑](#endnote-ref-3)
